# Genome Association Study for Visual Scores in Nellore Cattle Measured at Weaning

**DOI:** 10.1186/s12864-019-5520-9

**Published:** 2019-02-20

**Authors:** Luis Orlando Duitama Carreño, Matilde da Conceição Pessoa, Rafael Espigolan, Luciana Takada, Tiago Bresolin, Ligia Cavani, Fernando Baldi, Roberto Carvalheiro, Lucia Galvão de Albuquerque, Ricardo da Fonseca

**Affiliations:** 1 Animal Science Department, School of Agricultural and Veterinary Sciences, São Paulo State University (Unesp), Jaboticabal, São Paulo Brazil; 2 Animal Science Department, São Paulo State University (Unesp), Dracena, São Paulo, Brazil

**Keywords:** BayesC, Bayesian LASSO, Growth genes

## Abstract

**Background:**

Genome-wide association studies (GWAS) are utilized in cattle to identify regions or genetic variants associated with phenotypes of interest, and thus, to identify design strategies that allow for the increase of the frequency of favorable alleles. Visual scores are important traits of cattle production in Brazil because they are utilized as selection criteria, helping to choose more harmonious animals. Despite its importance, there are still no studies on the genome association for these traits. This study aimed to identify genome regions associated with the traits of conformation, precocity and muscling, based on a visual score measured at weaning.

**Results:**

Bayesian approaches with BayesC and Bayesian LASSO were utilized with 2873 phenotypes of Nellore cattle for a GWAS. The animals were genotyped with Illumina BovineHD BeadChip, and a total of 309,865 SNPs were utilized after quality control. In the analyses, phenotype and deregressed breeding values were utilized as dependent variables; a threshold model was utilized for the former and a linear model for the latter. The association criterion was the percentage of genetic variance explained by SNPs found in 1 Mb-long windows. The Bayesian approach BayesC was better adjusted to the data because it could explain a larger phenotypic variance for both dependent variables.

**Conclusions:**

There were no large effects for the visual scores, indicating that they have a polygenic nature; however, regions in chromosomes 1, 3, 5, 7, 14, 15, 16, 19, 20 and 23 were identified and explained a large part of the genetic variance.

## Background

With the advent of bovine genome sequencing [1], new information has become available for the prediction of genetic values through genomic selection (GS) and to locate regions or associated genes with phenotypes of interest through genome-wide association studies (GWAS) [2, 3]. In GWAS analyses, simple regression models are frequently utilized, however, this method has two limitations. The first limitation is the overestimation of the variance proportion explained by a marker, once it does not consider the existing linkage disequilibrium (LD) among markers [4]. The second limitation is the high rate of false positives when the population structure is not taken into consideration [5]. The use of Bayesian multiple regression models initially proposed for GS [6], such as Bayesian LASSO [7] and BayesC [8], overcomes these limitations.

The Bayesian approach differs with respect to a priori distribution for QTL (quantitative trait locus) effects. Bayesian LASSO assumes that there are few genes with large effects and many genes with small or no effects, whereas BayesC assumes that most SNPs (Single Nucleotide Polymorphisms) are not associated with phenotype, and only a small π portion has some effect on traits. The best model depends on the trait genetic architecture, which is unknown, mainly when the traits have not been extensively studied [9].

In cattle, association studies have been carried out for most economically important traits [10–13]. However, GWAS have not yet been conducted for morphological traits evaluated through visual scores in beef cattle, which have a high relevance to Brazilian cattle breeding. The utilization of these traits by producers as selection criteria is important for the identification of harmonious animals and its correlation with economic interest traits, mainly those for growth [14–16] and those that indicate carcass quality [17–19].

In dairy cattle, the results of some association studies for linear evaluations of type, which assess several morphological traits [20–23], are already available. In general, these studies did not find regions with large effects, showing that morphological traits have a polygenic nature; that is, they are controlled by multiple genes with small effects [5]. The distribution of QTL effects for traits is a factor that influences the prediction accuracy of genomic breeding values and this accuracy is greater when there are large effect QTLs [5, 24].

Due to the importance of visual scores for Brazilian cattle breeding, it is desirable that the regions involved in the genetic control of these traits have pleiotropic effects on other important economic traits and that there are large effect QTLs, resulting in greater genetic gains when utilized as selection criteria. This study aimed to identify genome regions associated with the traits of conformation (C), precocity (P) and muscling (M) visual scores, measured at the weaning of Nellore cattle, and to compare BayesC and Bayesian LASSO in a genomic association study.

## Methods

### Ethics statement

Data collection procedures were reviewed and approved by the Ethical Committee for Animal Care and Use (CEUA) of the São Paulo State University, UNESP - Jaboticabal, São Paulo, Brazil (protocol number: 18.340/16).

### Data set

Data were used from Nellore males and females, born between 2007 and 2011, belonging to two animal breeding programs, DeltaGen and Paint, and including more than 250 farms distributed across Brazil. Although the animals were from two distinct programs, they had a similar objective selection for weaning and yearling weight, and several sires were common to both programs, which were used for artificial insemination service. A previous GWAS analysis on birth weight revealed that the animals from these two breeding programs clustered in the same group (“cluster 1”) of a principal component analysis based on the genomic kinship coefficient [25].

Phenotypes for the visual scores were evaluated at weaning. Each contemporary group was assessed by a single technician. First, the whole herd was observed and characterized. Then, the average profile for each trait was used as a basis of comparison to attribute scores that varied from 1 to 5, where 5 was the highest expression and 1 was the smallest expression. Each visual score was defined as [26]: conformation (C) estimates the amount of meat on the carcass by length, body depth and muscle development; precocity (P) represents the ability of the animal to display the lowest acceptable degree of finishing with a low body weight, considering rib depth and fat deposition at the groin and tail of the animals at the time of evaluation; muscling (M) measures the amount of muscular mass, using as a reference the muscular development in the shoulder, foreleg, loin, rump and hind.

A total of 2021 females and 1416 males with records for C, P and M with genotypical information were utilized for the analyses. For genome association studies, the phenotype of visual scores and the deregressed breeding values (dEBV) were used as dependent variables for 2873 records after accounting for quality control and the smaller number of records for dEVBs (Table 1) as only animals with an accuracy over 0.60 were considered for inclusion.

**Table 1:** Number of records, according to the dependent variable, in genome association analyses for visual scores of conformation (C), precocity (P) and muscling (M)

Score	Dependent variable	Record			Average/Frequency*				
		Males	Females	Total	1	2	3	4	5
C	Phenotype	1977	896	2873	0.036	0.186	0.400	0.263	0.115
	dEBV	1266	601	1867			0.232		
P	Phenotype	1977	896	2873	0.040	0.164	0.355	0.279	0.162
	dEBV	1132	569	1701			0.293		
M	Phenotype	1977	896	2873	0.055	0.184	0.363	0.246	0.152
	dEBV	1118	553	1671			0.354		

*dEBV* deregressed breeding value; *frequency for phenotypic values and averages for deregressed breeding values, dEBV

Estimation of the breeding values (EBVs) of scores was done through a multi-trait threshold animal model, as described by [27]. Direct and maternal genetic, permanent environmental and residual effects were included as random effects, whereas contemporary group (farm, year of birth, management group at weaning, and sex) was used as a fixed effect and age at measurement was used as a co-variable (linear and quadratic effect). Genetic parameters and breeding values were estimated utilizing THRGIBBS1F90 software, which implements the Bayesian approach under a threshold model [28]. A total of 236,288 animals with phenotypes born from 1990 to 2012 and 300,484 animals included in the relationship matrix were used to estimate breeding values. EBV accuracy was calculated as described by [29] and deregressed breeding values were calculated using the methodology described by [30].

### Genotypic data

The animals were genotyped with Illumina BovineHD BeadChip (Illumina, San Diego, CA, USA), according to the manufacturer’s protocol. BovineHD BeadChip has 777,962 SNPs scattered throughout the genome with an average distance of 3.43 kb between markers. The inclusion criteria used to control genotype quality were: SNPs located in autosomes; call rate per SNP greater than 0.95; call rate per animal greater than 0.90; minor allele frequency greater than 0.05; *p*-value for Hardy–Weinberg equilibrium (HWE) test less than 1 × 10^− 5^ (extreme equilibrium deviations suggest potential genotyping errors). Highly co-related SNPs (r^2^ > 0.98) were excluded. Quality control was an interactive process that stopped when no SNP or sample were excluded, resulting in 309,865 SNPs for analyses that considered phenotype as the dependent variable, and 308,861, 308,561 and 308,481 SNPs for C, P and M, respectively, when the dependent variable was dEBV. Quality control and the input of missing genotypes were done with the snpStats package of R software [31].

### Association analyses

Bayesian approaches BayesC and Bayesian LASSO were utilized to estimate marker effects. Both Bayesian approaches differ in their a priori distributions, which are assumed for marker effects. In general, the models can be presented in matrix notation as:

1 $$ Y= X\beta + Z\alpha +e $$

where: *Y* is a vector n × 1 of phenotype or dEBVs for the visual score of C, P and M; *X* is an n × p matrix that relates *β* vectors of fixed effects with *Y*; *Z* is an n × k matrix of genotypes (0 for the first homozygote AA; 1 for the heterozygote AB or BA; 2 for the second homozygote BB) of k SNPs; *α* is a k × 1 vector of random coefficients of regression for SNPs (an effect of allele substitution) and *e* is a residual vector with a normal distribution *N* ∼ (0, *Iσ*^*2*^_*e*_), where *σ*^*2*^_*e*_ is the residual variance, considered unknown with a scaled inverse Chi-square distribution [32].

The difference between Bayesian approaches is the a priori marginal distribution assumed for α, which determines the variable selection and shrinkage in SNP effect estimates. For LASSO, the assumed distribution for effects is double-exponential. This distribution has a greater density at zero and thicker tails than a normal distribution, which causes an effect-dependent shrinkage because SNPs with small effects are regressed toward zero with greater shrinkage than the SNPs with large effects [7, 33]. Shrinkage level is controlled by the λ hyperparameter, which was inferred from data a priori using a Gamma distribution. For BayesC, the a priori effect distribution is a mixture at zero point mass and a normal distribution [8]. The proportion of SNPs with effects different from zero is controlled by the π hyperparameter, which was fixed at 0.01 instead of being inferred from the data since [34] found convergence problems when the π value was inferred from data. When score phenotype was considered a dependent variable, a threshold model with the probit function was utilized. The model assumes that there is a random variable subjacent to the observable phenotype, termed liability, which follows a standard normal distribution. The variance residual was fixed at 1 to make the estimation feasible. Fixed effects considered in the models were: contemporary group (formed by the management group at weaning, farm, birth year and sex) and weaning age, considered a co-variable. When dEVBs were the dependent variable, a linear model was utilized and only the average effect was considered a fixed effect in the model.

Analyses were carried out with the BGLR package of the software R [35], which implements Gibbs sampler to sample a posteriori parameter distributions. A total of 800,000 cycle chains were sampled. The first 200,000 were discarded as burn in and the remaining 600,000 samples were left for parameter inference.

### Association criterion

With the use of a high-density chip, QTL effects can be distributed across several SNPs that are in LD with QTL, resulting in non-significant individual SNP effects [36, 37]; therefore, SNPs were grouped in 1 Mb-long windows, overlapping every 100 kb, totaling 25,250 windows. The amount of SNPs per window varied from 1 to 336, with an average of 122 ± 25.2 SNPs. The genetic variance percentage, explained by each window, was the criterion used to identify windows associated with scores; it was calculated as follows:

2 $$ \%{\sigma}_j^2=\frac{\sigma_j^2}{\sigma_{SNP}^2}\ \frac{h^2}{h_{SNP}^2}100 $$

where: %σ^2^_j_ is the genetic variance percentage explained by window j; σ^2^_j_ is the genetic variance explained by window j; σ^2^_SNP_ is the genetic variance explained by SNPs; h^2^ is the trait heritability and h^2^_SNP_ is the phenotypic variance proportion explained by markers (marker heritability). σ^2^_j_ and σ^2^_SNP_ were calculated as the variance of genomic breeding values (GEBV) of each window or the whole genome. GEBV for animal *i* in window *j* was calculated as:

3 $$ {GEB\mathrm{V}}_{ij}={\sum}_{k=1}^K{X}_{ik}\widehat{\alpha_k} $$

where: *k* is the number of SNPs within window *j*; X_ik_ is the genotype of animal *i* for SNP k and α_k_ is the allele substitution effect for SNP k. For 1 SNP windows, this method is equivalent to 2p_k_(1 − p_k_)α^2^_k_ [38]. The Bayesian approach that explained the greater proportion of phenotypic variance was chosen to identify genome regions associated with the phenotype; the genes for the windows that explained values above 0.25% of the additive genetic variance were identified, and a candidate gene was proposed as responsible for the variance explained by the window.

### Gene identification

The database from the National Center for Biotechnology Information (NCBI) (http://www.ncbi.nlm.nih.gov/snp/), loaded with bovine genome version UMD 3.1, was utilized to identify genes within the windows. Gene classification with respect to biological function was done through the Database for Annotation, Visualization and Integrated Discovery (DAVID) [39], available at http://david.abcc.ncifcrf.gov/.

## Results

In general, the proportion of phenotypic variance explained by SNPs was smaller than trait heritability. Moreover, BayesC explained the largest phenotypic variance and was nearest to heritability, and among the dependent variables, phenotype was superior to dEBVs. Table 2 also shows the number of windows that captured 10% of the genetic variance; in this criterion, BayesC with phenotype was superior, as well.

**Table 2:** Proportion of phenotypic variance explained by BayesC and Bayesian LASSO and the number of windows needed to explain 10% of the genetic variance for the visual scores of conformation (C), precocity (P) and muscling (M)

Score	h^2^	Model	Dependent	σ^2^_SNP_	σ^2^_e_	h^2^_SNP_	10% σ^2^_a_
C	0.44	BayesC	dEBV	1.031	0.619	0.275 [0.625]	139
			Phenotype	0.503	1.00	0.335	87
		LASSO	dEBV	0.970	0.711	0.254 [0.577]	175
			Phenotype	0.412	1.00	0.292	101
P	0.43	BayesC	dEBV	0.992	0.735	0.247 [0.574]	172
			Phenotype	0.390	1.00	0.281	82
		LASSO	dEBV	0.824	0.921	0.203 [0.472]	198
			Phenotype	0.322	1.00	0.244	99
M	0.42	BayesC	dEBV	0.902	0.753	0.229 [0.545]	159
			Phenotype	0.416	1.00	0.294	86
		LASSO	dEBV	0.850	0.834	0.212 [0.504]	205
			Phenotype	0.355	1.00	0.262	104

dEBV = deregressed estimated breeding values; h^2^ = estimated heritability utilizing the threshold model; σ^2^_SNP_ = variance explained by SNPs; σ_e_^2^ = residual variance; h^2^_SNP_ = proportion of phenotypic variance explained by SNPs, values in brackets [] indicate the proportion related to additive genetic variance; 10% σ_a_^2^ = number of windows that capture 10% of the genetic variance

**Fig. 1:**
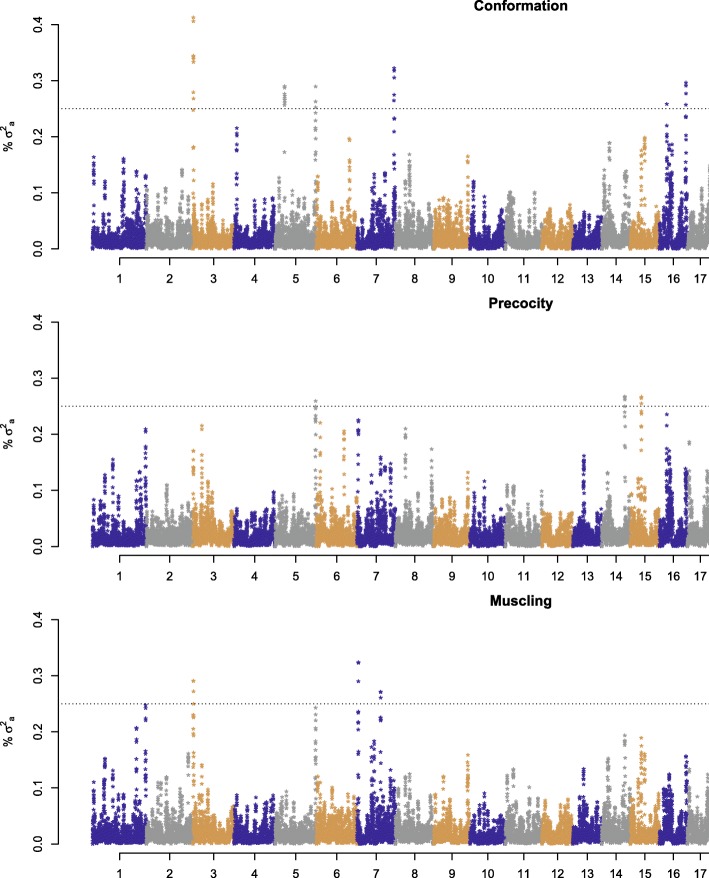
Percentage of genetic variance explained by windows in each chromosome using the Bayesian approach BayesC, considering phenotype as the dependent variable of visual scores for conformation, precocity and muscling. Dotted lines mark the 0.25% threshold of genetic variance explained by the windows

The identification of regions associated with visual scores was done through BayesC, utilizing phenotype as the dependent variable, because it was the Bayesian approach that best explained the proportion of phenotypic variance for all three visual scores. In Fig. 1 and Table 3, the windows that explained over 0.25% of the additive genetic variance for conformation, precocity and muscling are shown. Due to the great number of windows that were necessary to explain 10% of the genetic variance, and just a few windows exceeding the threshold of 0.25%, windows that were very close to the threshold were also included. For these windows, candidate genes responsible for the genetic variance explained by the window were proposed.

**Table 3:** Gene identification and percentage of genetic variance explained by windows associated with visual scores for conformation (C), precocity (P) and muscling (M), utilizing BayesC, considering phenotype as the dependent variable

BTA	Mb	% σ^2^_a_			Gene	Candidate	Description
		C	P	M			
1	155.9–156.9			0.25	1 proteins	TBC1D5	TBC1 domain family, member 5
3	0.4–1.4	0.41		0.29	10 proteins 1 tRNA 3 pseudogenes	CREG1 POU2F1	Cellular repressor of E1A-stimulated; POU class 2 homeobox 1
5	26.7–27.7	0.29			30 proteins	IGFBP6	Insulin-like growth factor protein 6
5	118.7–119.7	0.29	0.26	0.24	3 proteins 4 ncRNA	MAPK11 MAPK12	Mitogen-activated protein kinase 11 and 12
7	3.0–4.0			0.32	23 proteins 5 pseudogenes 2 ncRNA	LPAR2 NDUFA13	Lysophosphatidic acid receptor 2; NADH dehydrogenase 1 alpha, 13
7	59.0–60.0			0.27	9 proteins 2 ncRNA	PPP1R39	SH3 domain ring finger 2
7	108.8–109.8	0.32			2 proteins 1 tRNA 1 ncRNA	FBXL17	F-Box And Leucine-Rich Repeat Protein 17
14	67.5–68.5		0.27		11 proteins 1 tRNA 2 ncRNA	STK3	Serine/threonine kinase 3
15	31.7–32.7		0.27		5 proteins 1 miRNA	SORL1	Sortilin-related receptor, L(DLR class)
16	20.7–21.7	0.26	0.24		2 proteins 1 tRNA	ESRRG	Estrogen-related receptor gamma
16	80.7–81.7	0.30			7 proteins	TMEM9	Transmembrane protein 9
19	36.0–37.0		0.23		23 proteins 3 ncRNA 4 pseudogenes	WFIKKN2 ACSF2	Follistatin/kazal, immunoglobulin, kunitz and netrin domain; Hypothetical protein LOC768237
20	39.4–40.4			0.26	8 proteins 1 tRNA	C1QTNF3	C1q factor related protein 3
23	24.1–25.1		0.35	0.24	16 proteins 1 tRNA 2 ncRNA 2 miRNA 3 Pseudogene	GSTA3	Glutathione S-transferase, alpha 3
Total		1.87	1.62	1.87			

*BTA* bovine chromosome, *Mb* window position in megabases; %σ_a_^2^ = % of genetic variance explained by each window

A total of 190 genes were located in 14 associated windows with the phenotype of visual scores: 150 genes were codified proteins, 16 were ncRNA (non-codifying RNA), 3 were miRNA (microRNA), 6 were tRNA (RNA transporter) and 15 were pseudogenes. In the identification of candidate genes, only genes that codify proteins were considered. Thus, 18 candidate genes were identified and classified into four groups according to the function that they perform in the organism: basal and cellular metabolism (*TBC1D5, LPAR2, TMEM9, NDUFA13, GSTA3* and *FBXL17*), regulation and transcription of other genes (*CREG1, POU2F1, MAPK11, MAPK12, ESRRG* and *STK3*), lipid metabolism (*SORL1* and *ASCF2*) and genes related to growth and skeletal muscle (*C1QTNF3, PPP1R39, WFIKKN2* and *IGFBP6*). Furthermore, windows located in chromosomes 3 (0.4–14 Mb), 5 (118.7–119.7 Mb), 16 (20.7–21.7 Mb) and 23 (24.1–25.1 Mb) were associated with at least two scores (Table 3).

## Discussion

The difference between the proportion of phenotypic variance explained by SNPs and trait heritability, a difference known as lost heritability [40], can be explained by several factors, such as the lack of LD among markers and QTLs, interactions between variants, or interactions between genetics and the environment [41]. Another possible explanation could be the sample size, as it is necessary to increase the number of genotyped animals to capture small effect QTLs [42].

With respect to the Bayesian approaches used, BayesC had a better fit to the data as it was nearest to heritability and explained the largest phenotypic variance. In addition, phenotype was superior to dEBVs, although some authors have considered estimated breeding values (EBV) to be the best dependent variable for GWAS analyses [43] as they are the best estimate of genetic breeding value. The smallest phenotypic variance captured by the models that used dEBVs can be a consequence of a smaller number of animals used and a low estimation accuracy; once, 46% of genotyped animals only had one parent and 58% did not have progeny, resulting in 75% of EBVs with an accuracy ranging from 0.60 to 0.70.

The great number of windows needed to explain 10% of the genetic variance (Table 2) indicates that there are no large effect QTLs; therefore, the visual scores evaluated in Nellore cattle are influenced by many small effect genes, as reported for morphological traits in dairy cattle, in which large effect regions have not been identified, and the identified regions were not common among the studies [20–23].

The presence of genes related to basal and cellular metabolism, located in significant windows, is explained by the fact that these genes act on several tissues, affecting cell and body development as a whole and, therefore, contributing to general performance. These genes have an important pleiotropic effect because they are non-specific tissues that act on several body cells and metabolic pathways, which include: signaling among cells, protein synthesis and transportation, cell proliferation and survival, and transportation and formation of cell membrane and its receptors. The gene protein *NDUFA13* (also known as *GRIM-19*) is found in this gene group and it is a functional component of mitochondrial complex I, which is also involved in apoptosis and cellular energy production processes [44]; modifications in this gene could cause lower energy availability for cellular processes, decreasing tissue growth. Another example is gene *FBXL17*, which intervenes in protein recycling processes in part of proteosoma 26S, acting on several cellular processes. This gene is expressed at different levels in the skeletal muscle of bovines from 8 to 12 months old, indicating that it is associated with muscle development at this age [45].

Genes that regulate transcription can be divided into two subgroups: one formed by the genes *STK3, MAPK11* and *MAPK12*, which belong to the kinase family (serine/threonine-protein subgroup), and another that includes genes *POU2F1, CREG1* and *ESRRG*, whose main function is to modulate genetic expression (transcription factor).

Kinases are enzymes that catalyze protein phosphorylation through the transfer of the ATP phosphoryl group, changing protein configuration and resulting in its activation or inactivation. Kinases represent the largest protein family in eukaryotes and are involved in multiple cellular processes, including cellular signaling mechanisms and the activation of transcription factors [46]. The gene *STK3* (known in humans as *MST2*) acts as a repressor of genes involved in the fat deposition of Hanwoo bovines; the high expression levels of this gene have been associated with a low level of intramuscular fat, showing that this gene inhibits adipocyte proliferation [47]. The genes *MAPK11* and MAPK12 are part of the metabolic pathway p38 MAPK, which is a preserved mechanism of cellular response to a broad variety of extracellular signals, and is proposed as a regulator of cellular differentiation, proliferation and development [48]. As p38 MAPK is not tissue-specific, it would explain why this region was associated with the phenotype for all three visual scores.

In the second subgroup, genes *POU2F1* and *CREG1* are transcription factors that regulate the activity of multiple genes that act on several metabolic pathways; among them are genes involved in growth, proliferation and cellular differentiation processes. *POU2F1* was identified by [49] as one of the transcription factors that regulates the genes associated with the traits of growth and fat deposition in Iberian x Landrace pigs, whereas [50] found differences in the genic expression level of *CREG1* in the *psoas major* and *flexor digitorium* muscles of bovines, indicating that both genes act on the skeletal muscles and fat tissue. Gene *ESRRG* is a member of the receptor family related to estrogen (ESRR) that act as transcription activators for gene *PERM1*, involved in the energy metabolism of skeletal and cardiac muscle [51], making this gene associated with deposition in fat and muscle tissue.

Both genes involved in lipid metabolism were associated with the precocity score, which evaluates the animal’s capacity to deposit fat. Gene *SORL1* belongs to the low-density lipoprotein receptor family (LDLR) involved in cholesterol metabolism, whereas *ACSF2* is associated with lipid metabolism and adipocyte differentiation. These two genes have been associated with intramuscular fat deposition in swine and bovines [52, 53].

Out of the four genes related to growth, three were associated with precocity and muscling, and the miostatin gene (*GDF8*) region did not show an association with visual scores. The miostatin gene is important in muscular development and is one of the genes responsible for the presence of double musculature in breeds like the Belgian Blue and Limousin. Gene *PPP1R39* (also known as *SH3RF2*), like the miostatin gene, negatively regulates muscular tissue growth once low expression levels are associated with muscular hypertrophy. Studying fast and slow growth lines in broiler chickens [54], found out that a deletion had been fixed for gene *SH3RF2* in the fast growth line, and it was identified as the cause of the largest growth. In bovines [55], found out that this gene was under selection in the Blonde d’Aquitane breed, and was one of the genes responsible for the double muscle presence in animals.

Gene *WFIKKN2* is an inhibitory protein of genes *GDF8* (miostatin) and *GDF11*, involved in muscular development, leading the overexpression of this gene to produce muscular hypertrophy, as the miostatin gene negatively regulates muscular growth, as observed in rats and sheep [56, 57]. Genes *IGFBP6* and *C1QTNF3* positively regulate muscular growth in bovines and high expression levels are associated with cellular growth in skeletal muscles. Gene *C1QTNF3* is also involved in subcutaneous and intramuscular fat deposition [58, 59].

Windows located in chromosomes 3, 5, 16 and 23 were associated with at least two scores; this was expected since the genetic correlations among the traits were high, ranging from 0.80 to 0.92, indicating that the same genes were controlling the visual scores. The results of this study will help the selection process as the increase of favorable allele frequencies for identified genes will lead to greater genetic gains in visual scores.

## Conclusions

According to our results, visual scores have a polygenic nature because regions explaining a great percentage of the genetic variance were not found. However, DNA regions associated with visual scores were identified, containing genes that are part of important biological processes and molecular functions in body development.

## Abbreviations

C: Conformation
dEBV: deregressed breeding values
EBV: Breeding values
GEBV: Genomic breeding values
GS: Genomic selection
GWAS: Genome-wide association studies
HWE: Hardy–Weinberg equilibrium
LD: Linkage disequilibrium
M: Muscling
P: Precocity
